# The PPARα agonist fenofibrate attenuates disruption of dopamine function in a maternal immune activation rat model of schizophrenia

**DOI:** 10.1111/cns.13087

**Published:** 2018-11-21

**Authors:** Marta De Felice, Miriam Melis, Sonia Aroni, Anna Lisa Muntoni, Silvia Fanni, Roberto Frau, Paola Devoto, Marco Pistis

**Affiliations:** ^1^ Division of Neuroscience and Clinical Pharmacology, Department of Biomedical Sciences University of Cagliari Monserrato Italy; ^2^ Section of Cagliari Neuroscience Institute, National Research Council of Italy (CNR) Monserrato Italy

**Keywords:** dopamine neurons, electrophysiology, maternal immune activation, peroxisome proliferator‐activated receptor‐alpha, schizophrenia, sex differences

## Abstract

**Aims:**

Prenatal maternal immune activation (MIA) is associated with a risk to develop schizophrenia and affects dopamine systems in the ventral tegmental area (VTA), key region in the neurobiology of psychoses. Considering the well‐described sex differences in schizophrenia, we investigated whether sex affects MIA impact on dopamine system and on schizophrenia‐related behavioral phenotype. Furthermore, considering peroxisome proliferator‐activated receptor‐α (PPARα) expression in the CNS as well as its anti‐inflammatory and neuroprotective properties, we tested if PPARα activation by prenatal treatment with a clinically available fibrate (fenofibrate) may mitigate MIA‐related effects.

**Methods:**

We induced MIA in rat dams with polyriboinosinic‐polyribocytidylic acid (Poly I:C) and assessed prepulse inhibition and dopamine neuron activity in the VTA by means of electrophysiological recordings in male and female preweaned and adult offspring.

**Results:**

Poly I:C‐treated males displayed prepulse inhibition deficits, reduced number and firing rate of VTA dopamine neurons, and paired‐pulse facilitation of inhibitory and excitatory synapses. Prenatal fenofibrate administration attenuated detrimental effects induced by MIA on both the schizophrenia‐like behavioral phenotype and dopamine transmission in male offspring.

**Conclusion:**

Our study confirms previous evidence that females are less susceptible to MIA and highlights PPARα as a potential target for treatments in schizophrenia*.*

## 1 INTRODUCTION

Environmental factors, such as prenatal infections, can lead to aberrant brain development, emerging in pathological phenotypes. Clinical and preclinical studies have widely reported an association between maternal immune activation (MIA) and an enhanced risk of developing psychiatric disorders in offspring later in life.^1^

Prenatal exposure to polyriboinosinic‐polyribocytidylic acid (Poly I:C), a double‐stranded synthetic RNA that triggers an innate immune response, induces MIA in rodents by mimicking a viral infection and has been shown to induce schizophrenia‐like phenotypes in rodents.^2^ These phenotypes display behavioral abnormalities, including sensorimotor gating impairment as well as alterations in brain regions key in the neuropathology of psychoses, such as dopaminergic ventral tegmental area (VTA) or prefrontal cortex. While the pathophysiology of schizophrenia has not been conclusively determined, it is believed that dopamine transmission dysregulation importantly alters information processing in multiple domains and results in the global symptoms observed in schizophrenia.^3^ Neuroimaging studies in humans indicate that dopamine signaling is altered in both the mesocortico‐limbic^4^, ^5^ and nigrostriatal pathway. Hence, dysregulation of dopamine transmission in the striatum, especially in the rostral caudate, is considered an additional hallmark of schizophrenia.^3^, ^7^

Schizophrenia is a neurodevelopmental disorder that displays robust epidemiological sex differences, with males and females exhibiting different symptoms, disease prevalence, and treatment response. Indeed, it is well established that females are less susceptible to autism and to early onset schizophrenia than males.^8^ Nevertheless, most of the studies investigating the Poly I:C model have been limited to male offspring and findings in females are still sparse.^9^, ^10^

Several preclinical studies indicate that the schizophrenia‐related behaviors of adult offspring after MIA involve interactions between inflammatory events and prenatal brain development.^12^ Thus, pharmacological treatments with anti‐inflammatory properties during pregnancy might be effective to prevent or minimize the impact of MIA in offspring. Here, we hypothesize that, among different potential targets, the peroxisome proliferator‐activated receptors alpha (PPARα) might possess a therapeutic potential. PPARα are members of a family of nuclear receptor transcription factors that have been shown to play essential roles in diseases associated with inflammatory processes.^13^ Growing evidence suggests that gene expression modulated by PPARα might mitigate the inflammatory component that occurs in psychiatric diseases, such as depression^14^ and schizophrenia.^15^ Additionally, an association with the gene encoding for PPARα (*PPARA*) was found in patients with schizophrenia^16^ and the expression of these nuclear receptor genes was also downregulated in hair‐follicle cells from schizophrenia patients.^17^

On these bases, the first aim of our study was to investigate the impact of MIA on schizophrenia‐related behavior and dopamine transmission in rat female offspring versus males both at preweaning age and during young adulthood. In order to test the hypothesis of sex differences in the outcome of MIA, we designed this investigation as a follow‐up of our previous study, whereby, consistent with previous literature, we found that male offspring from Poly I:C‐treated dams show marked deficits in prepulse inhibition of startle reflex (PPI) and in dopamine transmission, such as higher baseline levels of dopamine in the nucleus accumbens (NAc) and reduced number and firing rate of spontaneously active VTA dopamine neurons.^18^ Although nigrostriatal dopamine transmission might be compromised in schizophrenia, in our study we focused on the VTA subregion as the vast majority of studies on animal models of schizophrenia rely on the examination of the mesolimbic dopamine pathway, and chose to analyze PPI, a behavior that is dependent on mesolimbic dopamine, as an index of psychotic‐like behavioral abnormalities.

The second aim of our study was to investigate whether fenofibrate, a PPARα agonist clinically used as lipid‐lowering medication, is able, when administered during pregnancy, to mitigate the effects of MIA in the offspring.

## 2 MATERIALS AND METHODS

All procedures were performed in accordance with the European legislation EU Directive 2010/63 and were approved by the Animal Ethics Committees of the University of Cagliari and by Italian Ministry of Health (auth. n. 658/2015‐PR). Animals were housed in groups of three to six in standard conditions of temperature (21 ± 1°C) and humidity (60%) under a 12‐h light/dark cycle (lights on at 7:00 am) with food and water available ad libitum. We made all efforts to minimize animal discomfort and to reduce the number of animals used.

### 2.1 Prenatal treatment

**Figure 1:**
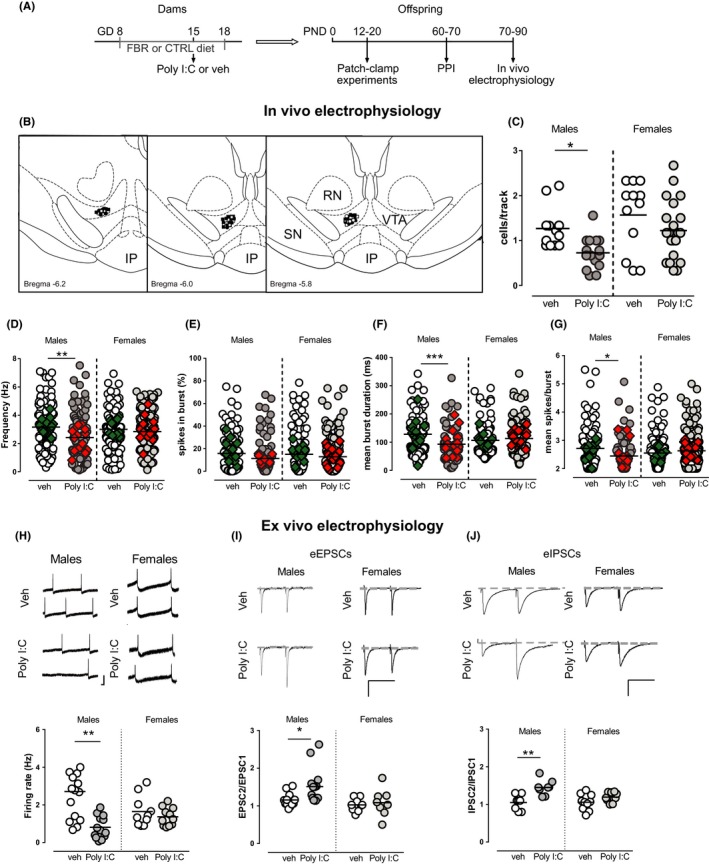
MIA impacts on VTA dopamine neuron activity in male but not female offspring. (A) Diagram representing the experimental protocol. Dams were fed with standard or 0.2% fenofibrate‐enriched diet from gestational day (GD) 8 to GD 18. At GD 15, a single iv injection of Poly I:C (4 mg/kg) or vehicle (sterile pyrogen‐free saline) was administered. Offspring underwent different experiments according to their postnatal age: patch‐clamp recordings were carried out between postnatal day (PND) 12‐20, prepulse inhibition of sensorimotor gating was tested at PND 60‐70, and in vivo electrophysiology experiments were performed at PND 70‐90. (B) Representative localization of recording sites of VTA putative dopamine neurons in vehicle (white dots for males and white squares for females) and Poly I:C (black dots for males and black squares for females) offspring, as verified by histological sections. RN, red nucleus; IP, interpeduncular nucleus, SN, substantia nigra pars reticulata. (C) Poly I:C offspring showed a reduced number of spontaneously active dopamine neurons. (D) Two‐way ANOVA yielded a significant interaction between sex and treatment for firing rate of dopamine cells. Hence, when compared with controls, the mean firing rate of VTA dopamine cells was decreased in males, as showed by the scatter plot. Burst duration (F) and mean spikes per burst (G) are impaired by MIA only in male offspring (two‐way ANOVA showed a significant interaction between sex and treatment). No significant difference was found for the percentage of spikes in burst (E) between sex or treatment. Superimposed colored diamonds show the averages for each individual rat. (H, I, J) Comparison of VTA dopamine cells from Poly I:C and controls in male and female offspring during preweaning age ex vivo. The graph shows individual firing rates of VTA dopamine neurons (H) recorded from male and female offspring from vehicle‐treated and Poly I:C‐treated dams. Each circle represents the mean firing rate of a 3‐min recording. Representative traces of action potential are displayed on top (calibration bars: 50 ms and 100 pA). No changes in paired‐pulse ratio (EPSC2/EPSC1) of AMPA EPSCs are observed between Poly I:C and vehicle female offspring (I), whereas a paired‐pulse facilitation is found in Poly I:C male rats as compared to controls. Representative traces of recordings from vehicle (top) and Poly I:C (bottom) rats are shown above graphs (calibration bars: 50 ms and 100 pA). Similarly, a paired‐pulse facilitation (IPSC2/IPSC1) has been observed in GABAA IPSCs of Poly I:C male offspring compared to vehicle rats, but not in females (J). Representative GABAA IPSCs from VTA dopamine neurons recorded in vehicle (top) and Poly I:C (bottom) rats are shown above graphs. *N* values are indicated in Table 1. Horizontal black lines represent means. Statistical analysis was conducted with two‐way ANOVA (sex and treatment as factors, see Table 1) and Sidak's multiple comparison test. Asterisks on graphs represent the results of the Sidak’s multiple comparison test: *P < 0.05, **P < 0.01, ***P < 0.001

Female Sprague Dawley rats (Envigo, Italy) were mated at the age of 3 months. The first day after the copulation was defined as gestational day 1 (GD 1). Dams were randomly assigned to two experimental groups: The first group received a control diet for the whole pregnancy, whereas the second group received a diet enriched with the PPARα agonist fenofibrate (0.2% w/w) ad libitum from GD 8 to GD 18*. *MIA was induced at GD 15, following the procedure described by Zuckerman et al.^2^ Dams were anesthetized with isoflurane 2% and a single dose of Poly I:C (4.0 mg/kg, iv) (InvivoGen, San Diego, CA, USA) or an equivalent volume of endotoxin‐free saline solution was administered in the lateral vein of the tail (see Figure 1A for a schematic representation of protocol). To assess the efficacy of Poly I:C injection, all pregnant rats were weighed for the first 3 days after the administration of either Poly I:C or saline to evaluate weight loss as underlined by previous investigations.^2^ After weaning, offspring were housed with littermates and maintained undisturbed until experiments. Subsequently, rats were randomly assigned to the experimental procedures and care was taken to avoid assigning more than three animals from the same litter to the same experimental group. In fact, for the experiments described here a total of 33 dams were utilized (15 were treated with vehicle and 18 with Poly I:C).

Each rat underwent only one experimental procedure, with the exception of those tested for prepulse inhibition, which were subsequently (following a recovery of at least 10 days) assigned to in vivo electrophysiology experiments.

Prepulse inhibition experiments were carried out in rats at postnatal day (PND) 60 to 70, whereas in vivo electrophysiology experiments were carried out at PND 70‐90. This age window, which corresponds to the late adolescence or young adulthood in humans, was selected as it is the most vulnerable age for the onset of schizophrenia.^8^ Moreover, studies on the ontogeny of MIA‐induced deficits showed that these are evident at PND 70.^19^, ^20^ Ex vivo electrophysiological experiments were carried out before weaning (PND 12‐20) to the aim of detect early abnormalities in dopamine neuron activity and synaptic properties.

### 2.2 Ex vivo and in vivo electrophysiological experiments

Whole‐cell patch‐clamp recordings from rat VTA dopamine cells were as described previously.^21^, ^22^ Extracellular single‐unit recordings from VTA DA neurons in anesthetized rats were as described previously.^18^, ^21^, ^23^ See Method S1 for more details.

### 2.3 Prepulse inhibition (PPI) of startle reflex

Startle and PPI were performed as previously described by Frau et al^24^ with slight modifications. See Method S1 for more details.

### 2.4 Statistical analysis

Statistical analysis is described in detail as Method S1.

## 3 RESULTS

In agreement with previous studies,^2^ rat dams underwent a significant weight loss in the 24 hours following Poly I:C systemic administration (−5.78 ± 1.6 g n = 18; vs controls +7.00 ± 1.5 g n = 13; *t*_(29)_ = 5.54, *P* < 0.0001, Student's *t* test; data not shown). This weight loss indicates that Poly I:C treatment induced a flu‐like syndrome in treated rats. However, Poly I:C treatment did not affect litter size (controls: 11.3 ± 1.2 pups, n = 13; Poly I:C: 12.0 ± 0.9 pups, n = 16, *P* = 0.64, Student's *t* test). Poly I:C treatment did not significantly affect abortion rate when compared with controls, as two dams from the control group (2/15) were excluded from the study as they interrupted their pregnancy, whereas two from the Poly I:C group (2/18) were excluded as offspring were not growing normally. Offspring were randomly selected for the experimental procedures, taking care that no more than three animals (one male and two females or two males and one female) from the same litter were assigned to the same experimental group or procedure (see Tables 1 and 2 for the number of litters used for each experimental group and procedure).

**Table 1:** This table shows the effect of vehicle or Poly I:C administration during pregnancy in male and female offspring. Statistical analysis was conducted with two‐way ANOVA (sex and Poly I:C treatment as factors) or Student's *t* test

	Females		Males		*P*
	Vehicle	Poly I:C	Vehicle	Poly I:C	
In vivo electrophysiology					
Cells/track	1.57 ± 0.23, n = 12	1.22 ± 0.14, n = 21	1.27 ± 0.13, n = 12	0.73 ± 0.09, n = 15	[Link](Poly I:C); [Link](sex)
Firing rate (Hz)	3.00 ± 0.13, n = 118	2.83 ± 0.11, n = 155	3.15 ± 0.16, n = 107	2.45 ± 0.17, n = 101	[Link](interaction)
Burst firing (%)	15.1 ± 1.8, n = 118	12.9 ± 1.4, n = 118	15.1 ± 1.7, n = 107	11.0 ± 1.7, n = 101	[Link](Poly I:C)
Spikes/burst	2.6 ± 0.1, n = 97	2.6 ± 0.1, n = 131	2.7 ± 0.1, n = 95	2.4 ± 0.1, n = 75	[Link](interaction)
Burst duration (ms)	106 ± 5.9, n = 97	112 ± 5.2, n = 131	1278 ± 8.2, n = 95	91.8 ± 6.7, n = 75	[Link](interaction)
No. of litters	N = 6	N = 13	N = 7	N = 8	
Ex vivo electrophysiology					
Firing rate (Hz)	1.65 ± 0.26, n = 10	1.37 ± 0.14, n = 11	2.67 ± 0.42, n = 15	0.81 ± 0.17, n = 13	[Link](interaction); [Link](Poly I:C)
EPSC2/EPSC1	1.03 ± 0.06, n = 9	1.08 ± 0.13, n = 8	1.16 ± 0.05, n = 13	1.51 ± 0.13, n = 14	[Link](Poly I:C); [Link](sex)
AMPA/NMDA ratio	0.65 ± 0.04, n = 6	0.56 ± 0.07, n = 6	0.44 ± 0.05, n = 8	0.34 ± 0.07, n = 9	ns
mEPSC frequency (Hz)	nd	nd	1.16 ± 0.11, n = 5	0.37 ± 0.03, n = 5	^§^
mEPSC amplitude (pA)	nd	nd	19.0 ± 2.58, n = 5	18.9 ± 3.13, n = 5	ns
IPSC2/IPSC1	1.06 ± 0.07, n = 10	1.19 ± 0.04, n = 9	1.05 ± 0.07, n = 8	1.45 ± 0.10, n = 6	*(interaction); [Link](Poly I:C)
mIPSC frequency (Hz)	nd	nd	0.93 ± 0.09, n = 6	0.45 ± 0.14, n = 6	^§^
mIPSC amplitude (pA)	nd	nd	28.1 ± 1.73, n = 6	28.3 ± 1.35, n = 6	Ns
No. of litters	N = 6	N = 6	N = 8	N = 8	
Sensorimotor gating					
Startle amplitude (AU)	700 ± 66, n = 17	700 ± 46, n = 16	1016 ± 78, n = 20	1112 ± 72, n = 27	[Link](sex)
Latency to peak (ms)	71.2 ± 2.0, n = 17	70.0 ± 1.7, n = 16	65.9 ± 1.2, n = 20	64.9 ± 0.9, n = 27	[Link](sex)
Habituation (IBR %)	138 ± 19, n = 17	111 ± 12.7, n = 16	144 ± 8.0, n = 20	154 ± 9.4, n = 27	[Link](sex)
PPI (%)	47.8 ± 3.7, n = 17	46.7 ± 4.5, n = 17	64.3 ± 2.2, n = 20	52.7 ± 3.4, n = 27	[Link](sex)
No. of litters	N = 9	N = 9	N = 13	N = 14	

Ns, not significant; nd, not determined.

* *P* < 0.05,

** *P* < 0.01,

*** *P* < 0.001,

**** *P* < 0.0001 (two‐way ANOVA);

^§^ *P* < 0.05,

^§§^ *P* < 0.01,

^§§§^ *P* < 0.001 (Student's t test).

**Table 2:** This table shows the effect of a control diet or a fenofibrate‐containing diet (0.2% w/w in food pellets) on the consequences of vehicle or Poly I:C administration during pregnancy in male offspring

	Control diet^a^		Fenofibrate (0.2% w/w)		*P* Two‐way ANOVA
	Vehicle	Poly I:C	Vehicle	Poly I:C	
In vivo Electrophysiology					
Cells/track	1.27 ± 0.13, n = 12	0.73 ± 0.09, n = 15	1.56 ± 0.24, n = 10	1.76 ± 0.23, n = 10	[Link](interaction)
Firing rate (Hz)	3.15 ± 0.16, n = 107	2.45 ± 0.17, n = 101	3.16 ± 0.16, n = 110	3.25 ± 0.15, n = 116	[Link](interaction)
Burst firing (%)	15.1 ± 1.7, n = 107	11.0 ± 1.7, n = 101	15.5 ± 2.0, n = 110	14.1 ± 1.8, n = 116	ns
Spikes/Burst	2.7 ± 0.1, n = 95	2.4 ± 0.1, n = 75	2.8 ± 0.1, n = 95	2.7 ± 0.1, n = 96	[Link](fenofibrate); [Link](Poly I:C)
Burst duration (ms)	128 ± 8.2, n = 95	91.8 ± 6.7, n = 75	131 ± 9.2, n = 95	119 ± 7.4, n = 96	[Link](Poly I:C)
No. of litters	N = 7	N = 8	N = 6	N = 6	
Ex vivo Electrophysiology					
Firing rate (Hz)	2.67 ± 0.42, n = 15	1.29 ± 0.36, n = 15	2.04 ± 0.33, n = 15	1.71 ± 0.21, n = 12	[Link](Poly I:C)
EPSC2/EPSC1	1.16 ± 0.05, n = 13	1.51 ± 0.13, n = 14	1.29 ± 0.1, n = 7	1.14 ± 0.16, n = 6	[Link](interaction)
IPSC2/IPSC1	1.05 ± 0.07, n = 8	1.45 ± 0.10, n = 6	1.05 ± 0.1, n = 14	1.08 ± 0.11, n = 9	[Link](fenofibrate)
No. of litters	N = 8	N = 8	N = 9	N = 7	
Sensorimotor gating					
PPI (%)	64.3 ± 2.2, n = 20	52.7 ± 3.4, n = 27	58.8 ± 3.3, n = 15	59.8 ± 2.4, n = 24	[Link](interaction)
No. of litters	N = 13	N = 14	N = 9	N = 14	

ns, not significant.

a Control groups are the same as in Table 1 and are reported here to facilitate comparison. See text for abbreviations. Statistical analysis was conducted with two‐way ANOVA (Poly I:C and fenofibrate treatment as factors).

* *P* < 0.05,

** *P* < 0.01 (two‐way ANOVA).

We previously showed that MIA disrupts behavior and affects dopamine transmission in male offspring.^18^ To investigate whether these effects are sex‐specific, we carried out, both in female and male offspring, in vivo and ex vivo electrophysiological recordings from VTA dopamine neurons and analysis of sensorimotor gating functions. The comparison between male and female offspring was carried out only in the group that received the control diet.

### 3.1 MIA disrupts activity of VTA dopamine cells in male but not female offspring: in vivo electrophysiology

We first determined whether estrous cycle influenced spontaneous dopamine neuron activity in female animals. Controls and Poly I:C female rats underwent vaginal smears before electrophysiological experiments and were staged according to their estrous cycle in estrus, proestrus, metestrus, and diestrus (Figure S1). The latter two groups were pooled in diestrus. By computing all electrophysiological parameter analyzed (cells/track, firing rate, burst firing) of dopamine cells according to the estrous stage (estrus, diestrus, and proestrus) and treatment (controls vs. Poly I:C), statistical analysis did not reveal any significant difference among estrous stages (Table S1). Therefore, data from all female rats were pooled independently of their estrous stage.

We next determined if Poly I:C prenatal treatment induced sex‐dependent effects on spontaneous activity of dopamine cells, by carrying out a population sample in the VTA. For these experiments, we utilized n = 12 vehicle‐treated and n = 15 Poly I:C‐treated male offspring, and n = 12 vehicle‐treated and n = 21 Poly I:C‐treated female offspring.

Analysis of the number of cells/track, which is an index of population activity of dopamine neurons, in the VTA (Figure 1B) revealed a significant effect of both treatment (*F*_(1, 56)_ = 8.05, *P* < 0.01) and sex (*F*_(1, 56)_ = 6.49; *P* < 0.05), but no interaction between sex and treatment (*F*_(1, 56)_ = 0.3920; *P* > 0.05) (Table 1, Figure 1C). On the other hand, two‐way ANOVA revealed a significant interaction between sex and treatment for several other electrophysiological parameters: firing rate of dopamine cells (*F*_(1, 477)_ = 3.86, *P* < 0.05, Table 1, Figure 1D), mean burst duration (*F*_(1, 394)_ = 10.16, *P* < 0.01, Table 1, Figure 1F), and the number of spikes in burst (*F*_(1, 394)_ = 4.582; *P* < 0.05, Table 1, Figure 1G). Analysis of the percentage of spikes in burst with two‐way ANOVA indicated that there was an effect of treatment (*F*_(1, 477)_ = 3.868; *P* < 0.05). As several neurons were recorded from individual rats and then grouped considering each cell as an independent replicate, a two‐way ANCOVA was carried out with sex and treatment as factors and individual subjects as covariate, to exclude that differences among individual rats had significant effects. The results indicated that individual subjects had no significant effect overall (effects of subjects analyzed with two‐way ANCOVA, firing rate *P* = 0.84; percentage of spikes in bursts *P* = 0.9; mean burst duration *P* = 0.65; number of spikes in bursts *P* = 0.66). Significance levels yielded by two‐way ANCOVA for factors or interaction between factors were the same as those obtained with two‐way ANOVA as indicated in Table 1.

Post hoc analysis (Sidak's multiple comparisons test), carried out when interaction between factors was significant, yielded a significant effect only between male controls and Poly I:C rats (Figure 1): dopamine neurons recorded from Poly I:C male offspring displayed a lower frequency, shorter bursts, and a lower number of action potential per burst, when compared with controls. No differences were detected by post hoc analysis between Poly I:C and control female rats (Figure 1C‐G). Our data indicate that dopamine cells in female offspring are less affected by MIA when compared with males.

### 3.2 Effect of MIA on VTA dopamine cells ex vivo in male and female offspring

Several studies have reported that Poly I:C administration during pregnancy leads to psychotic‐like behavioral abnormalities at adulthood without affecting rat behavior at preweaning age.^20^, ^25^, ^26^ Considered that, our next aim was to assess whether VTA dopamine neuron dysfunctions occurred later in the development, or early signs could be detected at a younger age. We carried out ex vivo whole‐cell patch‐clamp recordings from lateral posterior VTA dopamine neurons in horizontal slices from preweaned female and male offspring.

Two‐way ANOVA analysis of firing rate of dopamine cells recorded ex vivo from male and female rats revealed an effect of treatment (*F*_(1, 45)_ = 12.89; *P* < 0.001, Table 1, Figure 1H), and an interaction between sex and treatment (*F*_(1, 45)_ = 7.12, *P* < 0.05). Post hoc analysis (Sidak's multiple comparisons test) disclosed that male offspring displayed a marked reduction in the mean spontaneous firing rate of VTA dopamine cells recorded from rats exposed to Poly I:C, when compared with control males (Figure 1H), whereas in females the average spontaneous firing rate of VTA dopamine cells did not differ between Poly I:C and controls. Firing rate of dopamine neurons is the result of a complex balance between excitatory and inhibitory inputs and intrinsic membrane properties. An important factor in the regulation of VTA dopamine cell firing is the glutamatergic input from cortical and subcortical regions, which forms excitatory synapses on VTA dopamine cells.^27^ Therefore, we analyzed the property of excitatory synapses on VTA dopamine cells of female and male Poly I:C and control rats by comparing the changes in synaptic strength elicited by paired stimuli given at an interval of 50 ms (EPSCs2/EPSCs1 ratio). Two‐way ANOVA indicated an effect of both treatment (*F*_(1, 40)_ = 4.096; *P* < 0.05) and sex (*F*_(1, 40)_ = 7.498), but not of interaction (*F*_(1, 40)_ = 2.126, *P* > 0.05) (Figure 1I), on the change in synaptic strength elicited by paired stimuli. As both factors were significant, we ran a post hoc analysis that revealed difference only between male control and Poly I:C rats, as VTA dopamine cells of Poly I:C rats showed a paired‐pulse facilitation of AMPA‐mediated EPSCs, whereas no difference was detected between female groups (Figure 1I). No differences in AMPA/NMDA ratio were found between sex or treatment groups (two‐way ANOVA: interaction between sex and treatment; *F*_(1, 25)_ = 2.495, *P* > 0.05; Table 1). Properties of GABA synapses (IPSCs2/IPSCs1 ratio) differed between treatments (*F*_(1, 31)_ = 17.29; *P* < 0.01) but not between sexes, and the interaction between main factors was significant (*F*_(1, 31)_ = 4.18, *P* < 0.05) (Figure 1J). Post hoc analysis showed that properties of GABA synapses were different in male Poly I:C rats as compared to control rats. In fact, we observed a paired‐pulse facilitation in dopamine cells recorded from Poly I:C male rats. On the other hand, IPSCs2/IPSCs1 ratio was similar in female Poly I:C rats when compared with control females (Figure 1J).

Increases in the paired‐pulse ratio in males are predictive of either a reduced probability of neurotransmitter release,^28^ or a reduced function of postsynaptic receptors, or a combination of these. Therefore, we examined spontaneous miniature AMPA EPSCs (ie, mEPSCs) and GABA_A_ IPSCs (ie, mIPSCs) to detect changes in AMPA and GABA_A _receptor function, number, or both. In Poly I:C rats, we found a reduction in frequency of both mEPSCs (*t*_(8)_ = 6.95, *P* < 0.0001, Student's *t* test; Table 1) and mIPSCs (*t*_(10)_ = 2.83, *P* < 0.001, Student's *t* test; Table 1). Furthermore, no significant difference has been observed in the amplitude of mEPSCs (t_(8)_=0.04, *P* > 0.05, Student's *t* test; Table 1) and mIPSCs (*t*_(10)_ = 0.08, *P* > 0.05, Student's *t* test; Table 1). Altogether, the paired‐pulse protocol and the decreased frequency of spontaneous miniature events indicate a reduced probability of glutamate and GABA release in the VTA of Poly I:C rats. In fact, a reduction in frequency but not in amplitude is considered to reflect a presynaptic decreased probability of transmitter release.

### 3.3 Effect of MIA on prepulse inhibition of startle reflex in male and female offspring

PPI provides an operational measure of sensorimotor gating, a neurological process that filters irrelevant from salient information. PPI deficits are found in schizophrenia patients, and in rodents are induced by the administration of dopaminergic agonists and reversed by benchmark antipsychotics.^29^, ^30^ Therefore, we investigated whether MIA affected PPI in female and male offspring.

**Figure 2:**
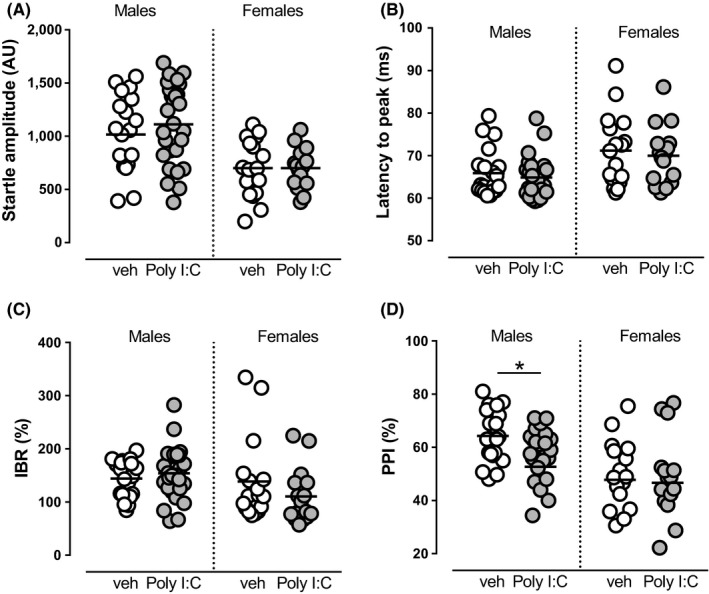
Effect of sex and Poly I:C treatment on startle reflex parameters. In both sexes, startle amplitude (A), latency to peak (B), startle habituation (C), and PPI (D) were strongly affected by sex (two‐way ANOVA, see Table 1), but no significant effect of MIA was detected as a main factor, as evoked by exposure of dams to Poly I:C, nor interaction between sex and treatment. *N* values are indicated in Table 1. Horizontal black lines represent means. Statistical analysis was conducted with two‐way ANOVA (sex and treatment as factors, see Table 1). Prepulses are indicated by the intensity corresponding to decibels above background noise. AU, arbitrary units; % IBR, percent inter‐block ratio

In females, experiments were analyzed pooling together subjects in different estrous cycle. Two‐way ANOVA showed an effect of sex for startle amplitude (*F*_(1, 76)_ = 24.91, *P* < 0.0001, Table 1, Figure 2A), latency to peak (*F*_(1, 76)_ = 13.24, *P* < 0.001, Table 1, Figure 2B), habituation (*F*_(1, 75)_ = 3,840, *P* < 0.05, Table 1, Figure 2C), and PPI (*F*_(1, 76)_ = 10.01, *P* < 0.01, Table 1, Figure 2D), but not a significant effect of prenatal treatment or interaction between factors. In fact, these results are due to significant differences between males and females in baseline value levels (see Table 1 and Figure 2A‐D) that make difficult to discern effects induced by Poly I:C treatments, if any. However, we carried out the following set of experiments only in male offspring, since when the effect of Poly I:C was analyzed separately within the same sex, we confirmed our previous observation that Poly I:C male rats display a disruption of PPI (*t*_(42.23)_ = 2.856, *P* < 0.01, Student's *t* test with Welch's correction, Figure 2).

### 3.4 The PPARα agonist fenofibrate protects male offspring from MIA‐induced alterations

As several detrimental effects induced by MIA were only evident in males, we evaluated the potential efficacy of a pharmacological treatment with a PPARα agonist to prevent neurodevelopmental aberrations solely in male rats. By measuring daily food intake, the oral intake of the PPARα agonist fenofibrate by dams was quantified in a subset of experimental subjects, resulting 119 ± 11 mg/kg per day (range 64‐196, n = 13).

**Figure 3:**
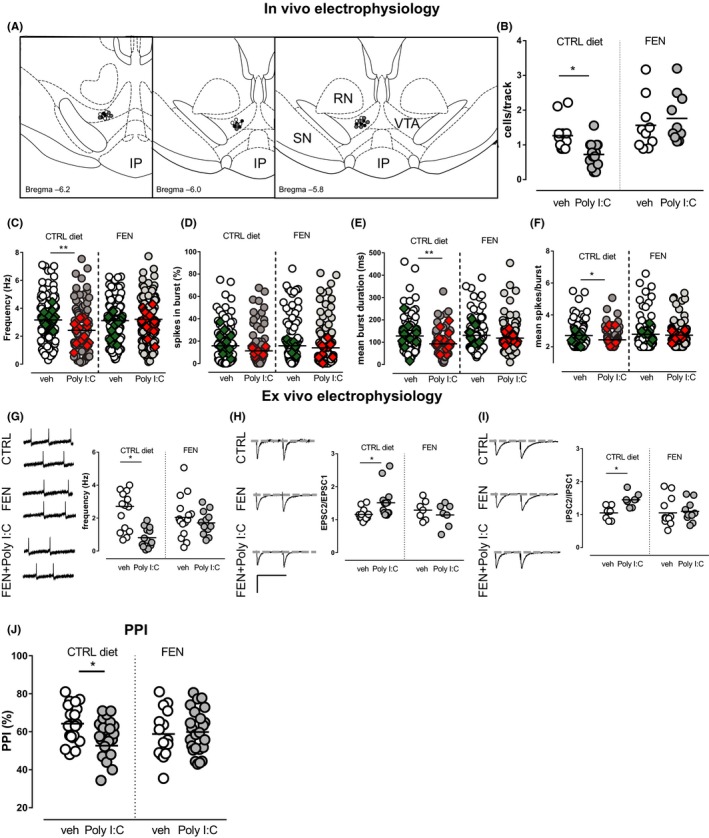
Fenofibrate prevented several MIA‐induced alterations on VTA dopamine neuron activity in vivo and ex vivo and attenuated sensorimotor gating deficits induced by MIA in males. (A) Sample localization of recording sites in controls (white dots), fenofibrate (gray dots), and Poly I:C + fenofibrate (black dots) rats, as verified by histological sections. RN, red nucleus; IP, interpeduncular nucleus; SN, substantia nigra pars reticulata. Fenofibrate administration, per se ineffective, prevented the Poly I:C‐induced decrease in the number of spontaneously active VTA dopamine neurons (B) and in the average firing rate (C). Moreover, the graphs show the effect of fenofibrate on alterations in the percentage of spikes in burst (D), mean burst duration (E), and mean number of spikes in bursts (F) induced by prenatal fenofibrate. Superimposed colored diamonds show the averages for each individual rat. (G‐I) Scatter plots showing that fenofibrate partially prevents MIA‐induced detrimental effects in preweaned rats. (G) Effect of fenofibrate on the mean firing rate of dopamine cells recorded from vehicle‐treated and Poly I:C‐treated offspring from mothers fed with a control diet or fenofibrate. AMPA EPSCs (H), but not GABAA IPSCs (I), paired‐pulse facilitation, resulting by prenatal exposure to Poly I:C, is prevented by fenofibrate diet during pregnancy (calibration bars: 50 ms and 100 pA). (J) Fenofibrate prevents PPI impairment induced by Poly I:C. Prepulses are indicated by the intensity corresponding to decibels above background noise. Control groups are the same as in Figure 1 and are reported here to facilitate comparison. *N* values are indicated in Table 2. Horizontal black lines represent means. Statistical analysis was conducted with two‐way ANOVA (Poly I:C and fenofibrate treatments as factors, Table 2) and Sidak's multiple comparison test. Asterisks on graphs represent the result of the Sidak's multiple comparison test: *P < 0.05, **P < 0.01

The timeline of the experiments, as well as the age of the animals, was exactly as stated in the section above and illustrated in Figure 1A. Exposure during pregnancy to fenofibrate prevented aberrations of VTA dopamine cell activity induced by MIA in the offspring, without inducing any significant effects per se. Specifically, two‐way ANOVA (factors: fenofibrate × Poly I:C) showed a significant interaction between factors for the mean number of cells per track recorded in VTA (cell/track index) (*F*_(1,43)_ = 4.93, *P* < 0.05, Figure 3A,B, Table 2) and for the firing rate of recorded dopamine cells (*F*_(1,429)_ = 6.286, *P* < 0.05; Figure 3C, Table 2). Post hoc analysis (Sidak's multiple comparisons test) revealed a significant effect of Poly I:C only in the offspring of dams fed with control diet and not in those from mothers fed with fenofibrate.

Although no change overall was shown for the percent of burst firing by two‐way ANOVA (Table 2, Figure 3D), fenofibrate‐enriched diet normalized the reduction in burst duration and spikes/burst observed in Poly I:C‐treated animals fed with the control diet (Table 2, Figure 3E‐F). For the mean spikes/burst parameter, two‐way ANOVA, however, did not reveal an interaction, but a main effect of both fenofibrate (*F*_(1, 356)_ = 4.855, *P* < 0.05) and Poly I:C (*F*_(1, 356)_ = 4.234; *P* < 0.05). Similarly, the analysis of the mean burst duration indicates a main effect of Poly I:C (*F*_(1, 357)_ = 8.706, *P* < 0.01) and a trend for the effect of fenofibrate (*F*_(1, 357)_ = 3.527; *P* = 0.06), but no interaction. As several neurons were recorded from individual rats and then grouped considering each cell as an independent replicate, a two‐way ANCOVA was carried out with fenofibrate and Poly I:C as factors and individual subjects as covariate, to exclude that differences among individual rats had significant effects. The results indicated that individual subjects had no significant effect overall (effects of subjects analyzed with two‐way ANCOVA, firing rate *P* = 0.83; percentage of spikes in bursts *P* = 0.62; mean burst duration *P* = 0.96; number of spikes in bursts *P* = 0.70). Significance levels yielded by two‐way ANCOVA for factors or interaction between factors were the same as those obtained with two‐way ANOVA as indicated in Table 2.

In light of these findings, we next investigated whether the protective effects of the PPARα agonist during pregnancy are evident early in life. Notably, two‐way ANOVA showed a significant main effect of Poly I:C treatment on firing rate of dopamine neurons (*F*_(1, 53)_ = 6.174, *P* < 0.05, Table 2, Figure 3G), but no interaction between factors. Properties of excitatory synapses were affected by the interaction between Poly I:C and fenofibrate treatments. In fact, for AMPA‐mediated EPSCs two‐way ANOVA revealed an interaction between treatments (*F*_(1, 37)_ = 4.781, *P* < 0.05), whereas the post hoc test showed that only Poly I:C rats of mothers fed with a control diet displayed an enhanced EPSCs2/EPSCs1 ratio and, therefore, a paired‐pulse facilitation (Table 2, Figure 3H). On the other hand, a main effect of fenofibrate (*F*_(1, 36)_ = 4.721, *P* < 0.05), but not of interaction or Poly I:C, was detected for GABA_A_‐mediated IPSCs (Figure 3I).

Finally, we investigated whether fenofibrate during pregnancy prevents PPI disruption in males. Two‐way ANOVA analysis showed an interaction between Poly I:C and fenofibrate treatments (*F*_(1,82)_ = 4.36, *P* < 0.05, Table 2, Figure 3J). Post hoc test (Sidak's multiple comparisons test) revealed that Poly I:C had a significant effect to decrease PPI only in the offspring from rats fed with control diet (Table 2, Figure 3J), whereas no difference was found between offspring from fenofibrate‐treated animals, indicating that fenofibrate was able to prevent PPI deficits induced by Poly I:C exposure (Figure 3J).

## 4 DISCUSSION

Our findings indicate that MIA, evoked by maternal exposure to Poly I:C, induces detrimental effects in offspring that are more severe in males than females, and that activation of the nuclear receptor transcription factors PPARα might represent a pharmacological strategy to prevent such an outcome.

In line with gender differences in the prevalence of many neuropsychiatric disorders, such as early onset schizophrenia^8^ and autism,^31^ we found that dopaminergic system of female offspring was less vulnerable to MIA, when compared to male littermates. Specifically, male littermates showed a reduction in spontaneous activity of VTA dopamine cells and a paired‐pulse facilitation of inhibitory and excitatory synapses onto dopamine cells. Effect of MIA on dopamine system was consistently demonstrated by previous studies. In particular, Vuillermot et al^19^ provided evidence that exposure to Poly I:C during early/middle gestation in mice leads to a complex pattern of age‐dependent structural abnormalities in mesoaccumbal and nigrostriatal dopamine systems. Thus, MIA might compromise the integrity of the developing fetal dopamine system, suggesting that prenatal exposure to immunological insults induces deficits in dopaminergic development.^19^

In fact, at the time of gestational Poly I:C exposure (GD 14 to GD 17), an inflammatory cytokine production might represent a window of higher vulnerability for the dopamine system, as studies have detected the maximal rise in dopamine neurons in the developing mesencephalon, that decreases again soon after (GD17 to GD 21).^32^ Interestingly, the density of TH‐positive fibers in projection regions such as the striatum presents a strong sex dimorphism, being higher in female rats.^33^ Additionally, male fetuses have been shown to possess lower mesencephalic TH immunoreactivity,^34^ and greater midbrain DA uptake^35^ in comparison with females. Male sex hormones have been involved in the normal postnatal reduction of male catecholamine levels, as gestational inhibition of aromatase and neonatal castration resulted in significantly higher cortical dopamine and DOPAC levels.^36^ Thus, sex dimorphism in prenatal development of dopamine neurons^33^ might underlie sex‐specific vulnerability of this system to prenatal insults, although the mechanism is still to be elucidated. Studies have highlighted that dysfunctions in dopamine transmission are not restricted to the meso‐corticolimbic pathway but might involve the hypothalamus as well. Hence, Kirsten et al^37^ showed that prenatal lipopolysaccharide (LPS) exposure induced sex‐dependent behavioral abnormalities (males are more affected than females) and reduced in a sex‐dependent manner hypothalamic dopaminergic metabolite levels (homovanillic acid, HVA) and the hypothalamic dopaminergic turnover rate (HVA/dopamine).^38^ Consistently, hypothalamus of Poly I:C‐treated mice displays significant global DNA hypomethylation.^39^ The hypothalamus displays developmental sex dimorphism and is involved in sexual differentiation and gonadal hormone synthesis^40^; thus, a contribution to sex‐related MIA‐induced deficits is likely.

Results from electrophysiological experiments in the present study are at odds with the reported increase in the number of TH‐immunoreactive cells in the VTA, TH‐positive terminals in the striatum,^19^, ^41^, ^42^ or increases in evoked striatal dopamine release ex vivo^2^ as well as augmented levels of dopamine in the lateral globus pallidus and prefrontal cortex.^42^ In fact, our examination of population activity of dopamine neurons in the VTA yielded a reduced number of spontaneously active cells together with a reduced firing activity. It must be pointed out, however, that the technique allows us to reveal only spontaneously active dopamine cells, so our findings do not exclude the possibility that the actual number of dopamine cells, if silent or firing too slowly to be detected by electrophysiology, could be higher. Indeed, elevated dopamine levels in the NAc might inhibit, via a long‐loop feedback, the firing activity of dopamine cells.

In our recent report,^18^ we showed that MIA ensuing Poly I:C exposure during gestation disrupts PPI in male adult rats. Here, when analyzing sex differences in the vulnerability of PPI response to MIA, we did not find an interaction between prenatal Poly I:C treatment and sex, as females showed control PPI parameters significantly different to males, and this latter might have masked any potential interaction. Other groups have previously reported sex differences in PPI and startle parameters on rats exposed to MIA.^43^, ^44^ However, the interaction between sex and MIA on the sensorimotor gating integrity should be considered with caution, given that the alterations of PPI observed in male and female MIA offspring appear to be dependent on the experimental setting and the timing of testing.^44^ On the other hand, Howland et al^45^ reported sensorimotor gating deficits at both shorter and longer prepulse intervals, irrespective of sex. Other studies show neither alterations in PPI parameters in both males and females^43^ nor remarkable PPI deficits in MIA‐exposed females but only later in life.^19^

Notably, we can exclude that estrous cycle affected our results in females. In fact, no differences related to the estrous cycle have been found in both Poly I:C and control offspring in neurophysiological and behavioral parameters examined.

We detected changes in dopamine neuron functions in preweaned rats: ex vivo electrophysiological recordings carried out from VTA dopamine cells showed that neurons from Poly I:C‐treated male offspring fire at a lower rate when compared with controls. This finding suggests changes in intrinsic membrane properties and/or ionic channel conductance, which might result in the observed reduction of spontaneous pacemaker‐like firing. Moreover, both excitatory and inhibitory afferents to dopamine cells displayed an increased paired‐pulse ratio in males only, that is, facilitation, at both synapses. This facilitation, together with the reduced frequency of mIPSCs and mEPSCs, is predictive of a reduced probability of GABA and glutamate release, respectively. Given that dopamine neuron's firing rate and pattern in vivo are regulated by the balance between excitatory and inhibitory inputs, any unbalance or subtle change in synaptic plasticity might affect the firing rate and pattern of dopamine cells. At this stage, the contribution to dopamine cell function by this altered equilibrium is neither clear, nor it is evident whether these subtle changes in intrinsic firing activity and/or afferent functions translate into behavioral abnormalities in preweaned male animals. It is likely that such alterations contribute to an endophenotype before adolescence, since studies report a number of behavioral abnormalities and cognitive impairments in adulthood but not in adolescent rodents,^2^, ^20^, ^25^, ^26^ as observed in schizophrenia. In this scenario, our challenge was to investigate on potential therapeutic targets that might attenuate the disruptions induced in MIA model. Here, we demonstrated that PPARα activation during pregnancy prevents impairments in the physiological activity of VTA dopamine neurons in vivo and ex vivo, and disruptions in sensorimotor gating evoked by MIA in male offspring. In recent years, interest in the physiological role of PPARα in the CNS has been growing, as these receptors might be involved in the neuropathogenesis, and therapy, of neurological and psychiatric disorders.^13^, ^46^, ^47^ PPARα displays a distinct pattern of expression in the CNS in neurons and glial cells,^48^ but the precise physiological role of PPARα in neurons is still unclear. As in the periphery, these receptors might be involved in lipid metabolism and energy balance in neurons and in protection from neuroinflammation. PPARα are also expressed by cells in the peripheral immune system, that is, T and B lymphocytes^49^ where it has been reported to determine the timing, sequence and magnitude of cytokines produced by activated T cells. PPARα are also expressed by macrophages, where decrease inflammation‐evoked activation and induce a decrease in the expression of NOS‐2 and proinflammatory cytokines.^50^ Our result is consistent with the anti‐inflammatory and neuroprotective properties of N‐acylethanolamines (NAEs), endogenous PPARs ligands, or fenofibrate in Parkinson's and Alzheimer's disease, epilepsy, and psychiatric disorders (ie, schizophrenia and depression).^13^, ^47^, ^51^, ^52^

The mechanisms of anti‐inflammatory and neuroprotective actions by PPARα in the CNS have not been fully clarified. One hypothesis is that PPARα activation negatively regulates the signaling of nuclear factor kappa‐light‐chain‐enhancer of activated B cells (NFκB) and the activator protein 1 (AP‐1),^54^ the expression of tumor necrosis factor‐α (TNF‐α)^55^ as well as the production of cytokines and interferons.^56^ In fact, in MIA models the stimulation of the TLR‐3 pathway induced by Poly I:C modulates the activation of NFκB and AP‐1 through mitogen‐activated protein kinases (MAPK) cascade,^57^ thereby leading to the synthesis of several proinflammatory cytokines, such as IL‐1, IL‐6, TNFα, and interferons.^57^

The activation of PPARα has been reported to increase the brain synthesis of oleoylethanolamide (OEA) and palmitoylethanolamide (PEA).^46^ These molecules display marked anti‐inflammatory properties in acute and chronic inflammation and sustain PPARα activity by eliciting a positive feedback.

Our findings confirm, in an experimental model, a reduced vulnerability in females reported in schizophrenia and autism in humans. Such results could contribute to unveil neurobiological mechanisms underlying sex differences in vulnerability to develop schizophrenia and other related disorders. In fact, the increasing knowledge of sex dimorphism might be important to plan a sex‐specific treatment for these complex disorders. Moreover, evidence is provided of protective effects of PPARα activation on neurodevelopmental aberrations induced by prenatal exposure to Poly I:C. Although fenofibrate is not recommended during pregnancy by the Food and Drug Administration and by the European Medicine Agency for potential hazardous effect on the fetus, no fenofibrate‐induced noxious effects in the development of fetuses have been observed in clinical^58^, ^59^ and preclinical studies,^60^ with only a mild fetotoxicity induced by very high doses.^60^ Elucidation about potential side effects of PPARα on fetal neurodevelopment is definitely compelling. Translational studies might be facilitated by the fact that strategies to achieve PPARα activation, beside fibrates, include supplements of specific dietary lipids that might be safer during pregnancy. The timing for optimal treatment with PPARα agonists is yet to be established and needs to be pondered considering safety issues of medications or of dietary lipids and the potential risks for the offspring, especially in vulnerable individuals.

## CONFLICT OF INTEREST

The authors declare no conflict of interest.
