# Supplementary material for: The PPARα agonist fenofibrate attenuates disruption of dopamine function in a maternal immune activation rat model of schizophrenia
Source: CNS Neurosci Ther. 2018 Nov 21;25(5):549–61. doi: 10.1111/cns.13087 (PMC6488881; doi:10.1111/cns.13087)
Supplement: Supplementary file 1 [file CNS-25-549-s001.pdf]

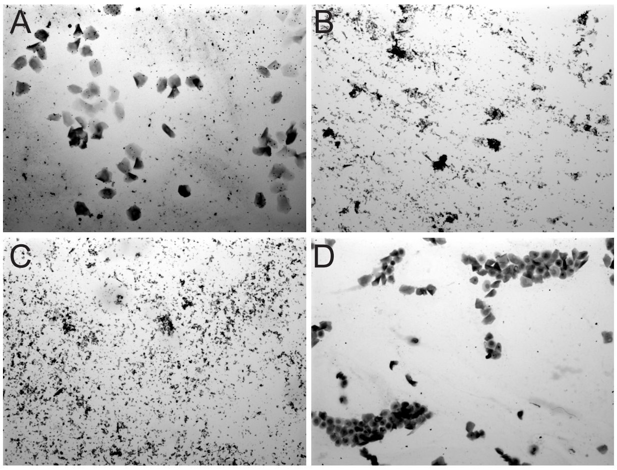

Supplemental Figure 1.
Representative photomicrographs of Giemsa's stained vaginal smears from female rats.
(A) estrus, (B) metestrus, (C) diestrus and (D) proestrus.
