# Supplementary material for: The PPARα agonist fenofibrate attenuates disruption of dopamine function in a maternal immune activation rat model of schizophrenia
Source: CNS Neurosci Ther. 2018 Nov 21;25(5):549–61. doi: 10.1111/cns.13087 (PMC6488881; doi:10.1111/cns.13087)
Supplement: Supplementary file 2 [file CNS-25-549-s002.docx]

**Supplemental Table 1**

|  | **Vehicle** | | |  | **Poly I:C** | | |  |  |
| --- | --- | --- | --- | --- | --- | --- | --- | --- | --- |
|  | **estrous** | **diestrous** | **proestrous** |  | **estrous** | **diestrous** | **proestrous** |  | **P** |
| cells/track | 1.50 ± 0.71  n=3 | 1.33 ± 0.35  n=6 | 2.11 ± 0.11  n=3 |  | 1.46 ± 0.45  n=4 | 0.80 ± 0.11  n=9 | 1.56 ± 0.22  n=8 | F(_1,27_)=2.01 | >0.05 |
| firing rate (Hz) | 2.74 ± 0.23  n=28 | 3.12 ± 0.20  n=51 | 2.99 ± 0.23  n=39 |  | 2.82 ± 0.20  n=35 | 3.10 ± 0.22  n=42 | 2.69 ± 0.15  n=78 | F(_1,267_)=0.22 | >0.05 |
| spikes in burst (%) | 15.64 ± 4.02  n=28 | 12.28 ± 2.18  n=51 | 18.26 ± 3.46  n=39 |  | 11.69 ± 2.83  n=35 | 17 ± 2.61  n=42 | 11.15 1.74  n=78 | F(_1,267_)=0.90 | >0.05 |

This table shows that stages of the estrous cycle do not significantly affect electrophysiological parameters of dopamine neurons recorded from female rats.
