# Supplementary material for: The PPARα agonist fenofibrate attenuates disruption of dopamine function in a maternal immune activation rat model of schizophrenia
Source: CNS Neurosci Ther. 2018 Nov 21;25(5):549–61. doi: 10.1111/cns.13087 (PMC6488881; doi:10.1111/cns.13087)
Supplement: Supplementary file 3 [file CNS-25-549-s003.docx]

**Supplemental methods**

***Electrophysiological experiments***

***In vivo recordings***

*In vivo* electrophysiological recordings were performed as described previously (1). At PND 70-90, male and female rats were anesthetized with urethane (1.3 g/kg, i.p.) and placed in the stereotaxic apparatus (Kopf, Tujunga, CA, USA) with their body temperature maintained at 37±1°C by a heating pad.

For the placement of a recording electrode, the scalp was retracted, and one burr hole was drilled above the parabrachial pigmented nucleus (PBP) of the posterior VTA (AP, 5.8-6.2 mm posterior from bregma, L, 0.4-0.6 mm lateral from midline) according to the Atlas of Rat Brain (2). We selected this subregion as it contains the largest density of dopamine cells as compared to the more medial portions of the posterior VTA.

Extracellular single-unit activity of dopamine neurons located in the VTA (V, 7.0-8.0 mm from the cortical surface) was recorded with glass micropipettes filled with 2% Pontamine sky blue (PSB) dissolved in 0.5 M sodium acetate (impedance 2.5–5 MΩ). The population spontaneous activity of VTA dopamine cells was determined in 6-9 predetermined tracks separated by 200 μm each other. Putative VTA dopamine neurons were selected when all criteria for identification were fulfilled: firing rate <10 Hz and duration of action potential >2.5 ms as measured from start to end (3). At the end of the experimental session, inhibition of spontaneous activity by DA receptor agonists and subsequent reversal by DA receptor antagonists was tested. Bursts were defined as the occurrence of two spikes at interspike interval <80 ms, and terminated when the interspike interval exceeded 160 ms (4). The electrical activity of each neuron was recorded for 2-3 min. Single-unit activity was filtered (bandpass 0.1–10000 Hz) and individual action potentials were isolated and amplified (Neurolog System, Digitimer, Hertfordshire, UK), displayed on a digital storage oscilloscope (TDS 3012, Tektronics, Marlow, UK) and digitally recorded. Experiments were sampled on-line and off-line with Spike2 software (Cambridge Electronic Design, Cambridge, UK) by a computer connected to CED 1401 interface (Cambridge Electronic Design, Cambridge, UK). At the end of recording sessions, DC current (15 mA for 15 min) was passed through the recording micropipette in order to eject PSB for marking the recording site. Brains were then rapidly removed and frozen in isopentane cooled to -40 °C. The position of the electrodes was microscopically identified on serial 60 μm sections stained with Neutral Red.

***Ex vivo recordings***

The preparation of VTA slices was carried out as described previously (5). Briefly, male and female offspring (PND 12-20) were anesthetized with isoflurane and euthanized. A block of tissue containing the midbrain was rapidly dissected and sliced in the horizontal plane (300 μm) with a vibratome (Leica) in ice-cold low-Ca^2+^ solution containing (in mM): 126 NaCl, 1.6 KCl, 1.2 NaH_2_PO_4_, 1.2 MgCl_2_, 0.625 CaCl_2_, 18 NaHCO_3_, and 11 glucose). Slices were transferred to a holding chamber with artificial cerebrospinal fluid (aCSF, 37°C) saturated with 95% O_2_ and 5% CO_2_ containing (in mM): 126 NaCl, 1.6 KCl, 1.2 NaH_2_PO_4_, 1.2 MgCl_2_, 2.4 CaCl_2_, 18 NaHCO_3_, and 11 glucose. Slices were allowed to recover for at least 1 hr before being placed, as hemislices, in the recording chamber and superfused with the aCSF (34-36°C) saturated with 95% O_2_ and 5% CO_2_. Cells were visualized with an upright microscope with infrared illumination (Axioskop FS 2 plus, Zeiss), and whole-cell patch clamp recordings were made by using an Axopatch 200B amplifier (Molecular Devices, CA). Current-clamp experiments and inhibitory postsynaptic current (IPSC) recordings were performed with electrodes filled with a solution containing the following (in mM): 144 KCl, 10 HEPES, 3.45 BAPTA, 1 CaCl_2_, 2.5 Mg_2_ATP, and 0.25 Mg_2_GTP (pH 7.2-7.4, 275-285 mOsm). All excitatory postsynaptic current (EPSC) recordings were carried out with electrodes filled with a solution containing the following (in mM): 117 Cs methansulfonic acid, 20 HEPES, 0.4 EGTA, 2.8 NaCl, 5 TEA-Cl, 2.5 Mg_2_ATP, and 0.25 Mg_2_GTP (pH 7.2-7.4, 275-285 mOsm). Experiments were begun only after series resistance had stabilized (typically 10-30 MΩ). Series and input resistance were monitored continuously on-line with a 5 mV depolarizing step (25 ms). Data were filtered at 2 kHz, digitized at 10 kHz, and collected on-line with acquisition software (pClamp 10.6, Molecular Devices, CA). Dopamine neurons from the lateral posterior VTA were identified according to the already published criteria (6): cell morphology and anatomical location (i.e. medial to the medial terminal nucleus of the accessory optic tract), slow pacemaker-like firing rate (<5 Hz), long action potential duration (>2 ms), and the presence of a large Ih current (>100 pA) (Johnson and North, 1992) that was assayed immediately after break-in, using a series of incremental 10 mV hyperpolarizing steps from a holding potential of -70 mV. A bipolar stainless steel stimulating electrode (FHC, USA) was placed 100 μm rostral to the recording electrode and was used to stimulate at a frequency of 0.1 Hz. Paired stimuli were given with an interstimulus interval of 50 ms, and the ratio between the second and the first PSCs was calculated and averaged for a 5 min baseline. Each slice received only a single drug exposure. The spontaneous miniature IPSCs (mIPSC) and EPSC (mEPSC) were collected in the presence of lidocaine (500 µM) and analyzed using Mini Analysis program (Synaptosoft). To accurately determine the mIPSC or mEPSC amplitude, only mIPSCs or mEPSCs that were >10 pA were accepted for analysis.

Drugs were applied in known concentrations to the superfusion medium. All the drugs were dissolved in DMSO. The final concentration of DMSO was <0.01 %.

***Prepulse inhibition of startle reflex***

At PND 60-70, male and female adult littermates were tested for prepulse inhibition of startle reflex (PPI). Startle and PPI were performed as previously described by (7). The apparatus used for detection of startle reflexes (Med Associates, St Albans, VT) consisted of four standard cages placed in sound-attenuated chambers with fan ventilation. Each cage consisted of a Plexiglas cylinder of 9 cm diameter mounted on a piezoelectric accelerometric platform connected to an analogue-digital converter. Two separate speakers conveyed background noise and acoustic bursts, each one properly placed to produce a variation of sound within 1 dB across the startle cage. Both speakers and startle cages were connected to a main PC, which detected and analyzed all chamber variables with specific software. Before each testing session, acoustic stimuli and mechanical responses were calibrated via specific devices supplied by Med Associates. On the testing day, each rat was placed in the cage for a 5 min acclimatization period consisting of 70 dB white noise background, which continued for the remainder of the session. Each session consisted of 3 consecutive sequences of trials (blocks). During the first and third block, rats were presented with only 5 pulse-alone trials of 115 dB. In the second block was delivered a pseudorandom sequence of 50 trials, including 12 pulse alone trials; 30 trials of pulse preceded by 74, 78, or 86 dB prepulses (10 for each level of prepulse loudness); and 8 no-stimulus trials, where only the background noise was delivered. Inter-trial intervals were selected randomly between 10 and 15 seconds while the inter-stimulus intervals were set at 100 ms. Startle response was based on the first positive wave that meets the minimum wave criteria and determined as mean startle amplitude of the pulse-alone trials relative to second block. Startle habituation across the two halves of the second block was evaluated as percent inter-block ratio using the following formula: (mean startle amplitude for first half of the second block/mean startle amplitude for the second half of the second block) × 100. Latency to startle was based on the first peak value across pulse-alone trials of the second block. “Arbitrary units” were calculated by the Med Associates apparatus software by proportionally converting the analog voltage signal recorded by the startle sensor (ranging from -10 to +10 V) to a digital unit, within a range of values between –2048 and +2048. The % PPI was calculated only on the values relative to the second block using the following formula: ([mean startle amplitude pulse alone trials - mean startle amplitude prepulse + pulse trials]/mean startle amplitude for pulse alone trials) × 100.

***Estrous cycle staging***

Vaginal smears were collected in female adult rats before experimental sessions. Samples were stained with Giemsa, and the cell morphology was microscopically examined to determine the estrous cycle stage (8). Four main phases were identified: estrous mainly displayed anucleated cornified cells, metestrus proportionally consisted of leukocytes, cornified and nucleated epithelial cells, diestrus mainly showed leukocytes, and proestrus displayed a predominance of nucleated epithelial cells (Supplemental Figure 1). The two phases metestrus (aka diestrus I) and diestrus (aka diestrus II) have been identified as diestrus.

***Data analysis and statistics***

Averaged data from different experiments are given as mean ± SEM. Data were checked for outliers and statistical significance was assessed using Student’s t-test, two-way ANOVA and two-way ANCOVA, where appropriate. Post-hoc multiple comparisons were made using the Sidak’s test. Data were analyzed using GraphPad Prism (San Diego, CA, USA). SPSS (Milan, Italy) was utilized for two-way ANCOVA. The significance level was established at P<0.05.

**References**

1. Luchicchi A, Lecca S, Melis M, De Felice M, Cadeddu F, Frau R, et al. Maternal Immune Activation Disrupts Dopamine System in the Offspring. Int J Neuropsychopharmacol. 2016;19(7).

2. Paxinos G, Watson C. The rat brain in stereotaxic coordinates. 7th edition London: Elsevier Academic Press. 2007.

3. Grace AA, Bunney BS. Intracellular and extracellular electrophysiology of nigral dopaminergic neurons--1. Identification and characterization. Neuroscience. 1983;10(2):301-15.

4. Grace AA, Bunney BS. The control of firing pattern in nigral dopamine neurons: burst firing. J Neurosci. 1984;4(11):2877-90.

5. Bonci A, Malenka RC. Properties and plasticity of excitatory synapses on dopaminergic and GABAergic cells in the ventral tegmental area. J Neurosci. 1999;19(10):3723-30.

6. Melis M, Scheggi S, Carta G, Madeddu C, Lecca S, Luchicchi A, et al. PPARalpha regulates cholinergic-driven activity of midbrain dopamine neurons via a novel mechanism involving alpha7 nicotinic acetylcholine receptors. J Neurosci. 2013;33(14):6203-11.

7. Frau R, Bini V, Pes R, Pillolla G, Saba P, Devoto P, et al. Inhibition of 17alpha-hydroxylase/C17,20 lyase reduces gating deficits consequent to dopaminergic activation. Psychoneuroendocrinology. 2014;39:204-13.

8. Marcondes FK, Bianchi FJ, Tanno AP. Determination of the estrous cycle phases of rats: some helpful considerations. Braz J Biol. 2002;62(4A):609-14.
